# An exploration of how meal preparation activities relate to self-rated time pressure, stress, and health in Canada: A time use approach

**DOI:** 10.1016/j.ssmph.2021.100818

**Published:** 2021-05-21

**Authors:** Michael J. Widener, Linda Ren, Chloe C. Astbury, Lindsey G. Smith, Tarra L. Penney

**Affiliations:** a Department of Geography and Planning, University of Toronto – St. George, 100 St. George St. Toronto, ON, M5S3G3, Canada; b School of Global Health, York University, 4700, Keele Street, Toronto, ON, M3J 1P3, Canada

**Keywords:** Meal preparation, Time pressure, Self-rated health, Time use survey, Canada

## Abstract

While previous work has provided a foundation for understanding the importance of the links between time use and diet, there has been little done to link time use to health outcomes. In this study, time use and self-rated health variables from the 2015 Time Use Cycle of Statistics Canada's General Social Survey are used to explore whether there are direct associations between time spent on meal preparation and health for Canadian Adults. In addition, this paper uses respondents' sequences of activities data from a time use diary to provide novel findings about the context of activities that precede and follow meal preparation. Proportional odds and logistic regression models are computed and show that there are significant relationships between spending more time on meal preparation and improved mental health and lower levels of stress. More time on meal preparation is also linked to general feelings of having less time. The analysis of activities preceding and following meal preparation activities demonstrates that individuals with different levels of self-rated stress or feelings of having extra time have significantly different activity sequence distributions (e.g., those reporting higher levels of stress are more likely to participate in chores and care activities). Exploring activity sequences related to meal preparation provides a first step in furthering the research community's grasp of the causal relationship between food-related time use and health and well-being outcome variables. Ultimately, this paper builds on the past literature on time use and meal preparation by establishing direct links between time spent on meal preparation activities, self-rated health and time use variables, in addition to offering insights into what activities surround this important activity via a novel sequence analysis.

## Highlights

• More time spent on meal preparation linked to better mental health and less stress.
• More time spent on meal preparation also linked to feelings of having less time.
• Activities adjacent to meal preparation differ by self-reported health and time use.

## 1 Introduction

Meal preparation, defined for this paper as the act of assembling a meal or snack for immediate or later eating, is a key component in the consumption of home-cooked meals. Numerous studies have shown that meals prepared at home are more nutritious (Mills, White, et al., 2017b) and are hypothesized to lead to positive downstream health impacts. As an example, a recent cross-sectional study from the United Kingdom found that more frequent consumption of home-cooked meals was associated with greater intake of fruits and vegetables, higher levels of plasma vitamin C, and lower percentages of excess body fat (Mills, Brown, et al., 2017a).

While evidence points to the benefits of home-prepared meals, researchers have also noted the additional burden such meals can place on a person or family. Compared with “convenience” meals that have been pre-prepared elsewhere, preparing meals from scratch at home requires extra labour, planning, shopping, and cleaning. This additional work can contribute to feelings of time scarcity (Celnik et al., 2012). The drivers of food-related feelings of time scarcity are multidimensional (Jabs & Devine, 2006), and include increasing numbers of dual-income households and changes in food retail environments and marketing. Poverty, in particular, can intensify feelings of time scarcity, as the ability to “buy time” by hiring services (e.g., childcare or grocery delivery) are not available (Jabs & Devine, 2006; Venn & Strazdins, 2017). Time scarcity can lead to time-saving behaviours including the purchase of convenience foods (Celnik et al., 2012), and is argued to be an important driver of overall health and well-being (Strazdins et al., 2011), with past studies showing links to negative mental health outcomes (Zuzanek, 1998).

Research on time spent on meal preparation dates back to the 1980s, with work focusing on parents and particularly mothers. Goebel and colleagues produced multiple studies that explored how family dynamics influenced time spent on food preparation and where meals are consumed. For example, one of their papers, using a sample of families from Wisconsin, demonstrated that households with employed mothers spent less time on food preparation and consumed more meals out of the home (Ortiz et al., 1981). In a second paper, with data from mothers living in 11 states across the USA, the authors showed time spent on meal preparation and dishwashing significantly varied by the age of their children and employment status (Goebel & Hennon, 1983). More recently, Jabs and colleagues (Jabs et al., 2007) expanded on this line of work by interviewing 35 employed, low-wage mothers about their strategies for carving out time for food and meals. They found that many of the interviewees expressed feelings of time scarcity and prioritized making time for their children's meals over their own.

Past work in this domain which has not focused explicitly on parents is more limited. However, three recent studies have shown that the links between food-related time use and dietary patterns for the general population is worthy of further examination. In a study using cross-sectional data from the Seattle Obesity Study, researchers examined how food consumption patterns are linked to time spent on food-related behaviours such as preparation, cooking, and cleaning (Monsivais et al., 2014). The authors reported that indicators of higher diet quality, including more consumption of fruits and vegetables and less money spent on food away from home, were associated with more time spent on meal preparation, cooking, and cleaning. Meanwhile, a study that examined six cross-sectional time-use surveys coupled with dietary surveys from 1965 to 2008 from the USA found secular declines in daily energy consumed from home food sources in tandem with declines in time spent preparing food (Smith et al., 2013). Furthermore, while lower-income individuals increased their time spent on preparing food between 1992 and 2008, they saw the largest decrease in the proportion who participated in meal preparation activities across the study period. In addition, a study by Astbury and colleagues (Astbury et al., 2020) analysed adults in the United Kingdom who spent various amounts of time on ‘food work’ (food preparation and other activities linked to eating) using data from a cross-sectional 2014-15 time-use survey. Their results indicate that spending more time on food work is linked to reductions in time spent sleeping and eating. Additionally, the authors reported that women experienced further reductions in time spent on activities such as socializing.

The previously described work provides a foundation for understanding the importance of the links between time use and diet, but there has been little done to link time use to health outcomes. Therefore, it is important to add to the literature on time use, time scarcity, and health, and in particular, to investigate whether specific types of time use are associated with health outcomes. Given the established links between both diet and health, and time use and diet, this paper explores if time spent on the activity of meal preparation is significantly related to a range of health outcomes.

Using data from a representative sample of Canadian adults, collected from the Statistics Canada 2015 General Social Survey Time Use Cycle (Turcotte et al., 2017), this study seeks to address gaps in the existing literature. Self-rated health variables (encompassing general health, mental health, and stress), along with self-rated sentiment towards time (specifically, feeling rushed or feelings of having extra time), are used to explore whether there are direct associations between time spent on meal preparation and health. In addition to these associations, data on respondents' sequences of activities from a time use diary provide novel findings about the context of activities that precede and follow meal preparation. This additional analysis is important, because the amount of time spent on meal preparation could be affected by, or affect, the time available to perform other activities throughout the day. By offering insights into what activities surround meal preparation activities via a novel sequence analysis, this study aims to provide further explanation to the research community's understanding of the relationship between food-related time use and health and well-being.

## 2 Material and methods

### 2.1 Data source

Data were used from the 2015 General Social Survey (GSS) on Time Use, which was collected over a one-year period between April 2015 and April 2016 (n = 17,390). Non-institutionalized residents who were 15 years and older and lived in one of ten Canadian provinces (excluding the territories of the Yukon, Northwest Territories, and Nunavut) were eligible to participate in the survey. Participants were contacted over the phone using the stratification plan described by Statistics Canada in the GSS – Time Use 2015 Public Use Microdata File User Guide (Turcotte et al., 2017). The survey is weighted to reflect age and sex distributions across the included provinces.

Interviewers worked through a series of modules with participants, where information including participants' demographics, household context, general perceptions of time use, well-being, and employment was collected. In addition, a detailed account of participants’ days starting at 4:00 a.m. was recorded by interviewers in the time use diary module. Participants described the sequence of their primary activities over a one-day period in 10-min intervals, including how long the activity lasted, their location, who they were with, and if any simultaneous activities occurred alongside the primary one. These responses were coded (see Appendix A of the GSS - Time Use 2015 Public Use Microdata File User Guide (Turcotte et al., 2017)), provided as a sequence for each user in an “episode” file, and then used to generate summary variables describing the cumulative time and count of activity episodes of various activities (e.g. total time spent eating would be the sum of the duration of all eating activities over the course of the day, while the count would be the sum of the number of eating activities, regardless of their duration). Derived time use diary variables (described in section 2.3) were grouped with responses to questions from the other aforementioned modules in a “main” file.

### 2.2 Included sample

The analytical sample for this paper included respondents aged 25–64 years (n = 11,254) who provided useable data for the variables used in the analyses (i.e., no replies of “don't know”, “refusal”, or “not stated”). Respondents in the ‘15 to 24,’ ‘65 to 74,’ and ‘75 and older’ age groups were removed in order to focus on working age adults.

### 2.3 Methods for the regression analysis

#### 2.3.1 Exposure and controls

The independent variables used in the models were the total duration of meal preparation activities (in 10-min intervals) and the count of meal preparation activities. Age group in years (1 = 25–34, 2 = 35–44, 3 = 45–54, 4 = 55–64), sex of respondent (1 = female, 0 = male), before tax household income (1 = $0-$19,999, 2 = $20,000-$39,999, 3 = $40,000-$59,999, 4 = $60,000-$79,999, 5 = $80,000-$99,999, 6 = $100,000-$119,000, 7 = $120,000-$139,999, and 8 = $140,000 or more), children in home (1 = children of the respondent are living at home, 0 = no children living at home), and working more than 30 h per week (1 = working >30 h, 0 = working <= 30 h) were included as controls.

#### 2.3.2 Outcomes

The outcomes modeled included two variables related to general perceptions of time use and three related to self-rated health. For the dependent time use variables, feeling rushed and feelings of having extra time are included, with both using the same Likert scale representing frequency (1 = every day, 2 = a few times a week, 3 = about once a week, 4 = about once a month, 5 = less than once a month, and 6 = never). The three self-rated health variables used were self-rated health (1 = excellent, 2 = very good, 3 = good, 4 = fair, and 5 = poor), self-rated mental health (1 = excellent, 2 = very good, 3 = good, 4 = fair, and 5 = poor), and self-rated stress (1 = not at all stressful, 2 = not very stressful, 3 = a bit stressful, 4 = quite a bit stressful, 5 = extremely stressful). For the purposes of this study, the self-rated health and mental health variables were recoded as binary variables (1 = excellent/very good/good, 0 = fair/poor) as was the self-rated stress variable (1 = extremely/quite a bit stressful, 0 = not at all/not very/a bit stressful). This recoding was done after the exploratory data analysis phase where the authors identified clear threshold effects when comparing the health-related variables to duration and count of meal preparation activities. The recoding strategy employed here mirrors the practice of other studies working with similar variables (Bratter & Gorman, 2011; Emerson et al., 2014).

#### 2.3.3 Analysis

Given the ordinal and binary nature of the response variables, proportional odds (for perception of time use variables) and binary logistic (for self-rated health variables) regression models were computed using the provided person weights and the ‘survey: analysis of complex surveys samples’ package (Lumley, 2004) in R (R Core Team (2020), 2020). The survey package provided a seamless way to conduct analyses of surveys with complicated sampling and weighting designs and provided various modeling tools (e.g., survey-weighted generalized linear models).

### 2.4 Methods for the sequence analysis

The previously described regression models aimed to establish any general associations between meal preparation duration or frequency with the outcome variables of perceptions of time use and self-rated health or stress. However, a further investigation of the ordering of activities surrounding meal preparation can provide insights into how this activity sequencing might vary for individuals with different perceptions of time use or self-rated health.

To analyze the sequence of activities surrounding meal preparation, the 18 high-level categories described in Appendix A of the GSS – Time Use 2015 Public Use Microdata File User Guide were reduced to nine categories, referred to herein as “super-activities.” This was done to increase the interpretability of the sequence analysis by deemphasizing activity types that were not very common prior to or following meal preparation (e.g., sports). The nine super-activity categories used are as follows:

1. meal preparation
2. sleeping, napping, resting, own personal care
3. eating or drinking
4. travel and going from place to place
5. paid work activities, study
6. household chores or maintenance, caring for children, teenagers, adults, those in other households
7. shopping for goods or services
8. socializing; community, civic, religious or organizational activities; sports, exercise or time spent outdoors, leisure
9. none, other, unencodable

The initial GSS data labeled “caring for children from your household,” as different from “caring for a teenager, caring for adults, and caring for those in other households.” These activities were merged with “household chores” as they related to labor occurring within the home. For super-activity “1,” sleeping was merged with “own personal care.” For super-activity “7,” outdoor, leisurely, and community activities were combined as one. Meal preparation activities were extracted from their initial high-level categories and treated as their own super-activity.

**Fig. 1:**
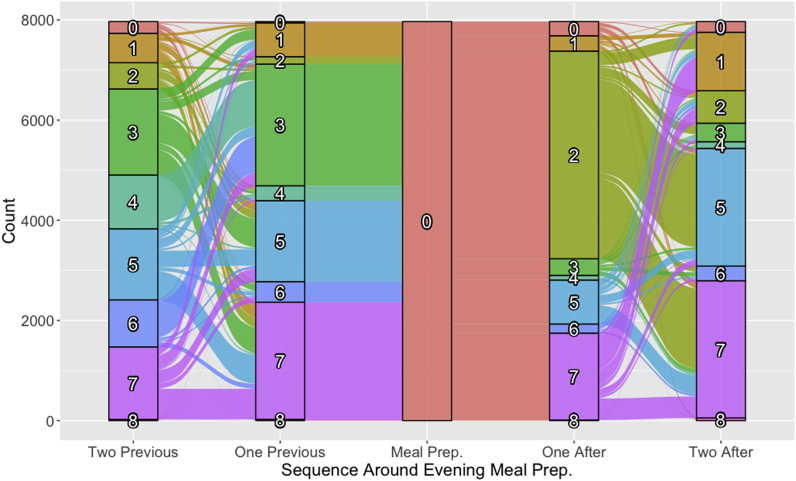
A Sankey diagram showing the flow of activities preceding and following evening meal preparation. Category numbers relate to super-activities where 0 is meal preparation, 1 is sleeping, napping, resting, own personal care, 2 is eating or drinking, 3 is travel and going from place to place, 4 is paid work activities, study, 5 is household chores or maintenance, caring activities, 6 is shopping for goods or services, 7 socializing or comm; civic religious/organizational activities; sports, exercise/outdoors, leisure, and 8 is none, other, un-codable.

After establishing these super-activity categories, an R script was used to determine every instance of a meal preparation activity in respondents’ time-use diaries that occurred between 3:00 p.m. and 9:00 p.m. This time period is used in order to focus on evening meals as a unique case, as morning and noon-time meals may have different patterning. With the meal preparation activities identified, the next preceding or succeeding super-activity adjacent to meal preparation was identified using an iterative search. This step was repeated to find the second-order adjacent preceding or succeeding super-activity, resulting in a total activity sequence length of five, with the third activity always being meal preparation. Note that because there are often multiple activities within each super-activity category (e.g., socializing and sports are both in category 7), super-activity categories may be adjacent to themselves in the presented Sankey diagram (Section 3.2, Fig. 1).

With these sequences built, an aggregate-level analysis of activities undertaken by Canadian adults before and after meal preparation was performed. Using the outcome variables described in section 2.3.2, differences between respondents with differing perceptions of time use and self-rated health were examined using chi-square tests.

## 3 Results

Weighted means and percentages of the variables used in the models are presented in Table 1. On average, respondents spent about 48 min on meal preparation activities and have slightly more than one meal preparation activity between 3:00 p.m. and 9:00 p.m. Respondents are on average between the 25–34 and 35–44 year old age group and have a household income close to $80,000-$99,999 CAD per year. Slightly more than half the respondents are female, while just under half have children living at home, and approximately 72% work 30 h per week or more.

**Table 1:** Summary statistics for variables used in regression models. For full variable categories and descriptions, please refer to Section 2.3. Note, meal preparation duration in tens of minutes is used in the regression models to help with the interpretation of reported odds ratios.

	Weighted Mean	S.E.
Meal Preparation Duration (min.)	48.295	0.8243
Count of Meal Prep. Activities	1.0508	0.0135
Age Group (1 = 25–34, 4 = 55–64)	2.5007	0.0144
Income Group (1 < $20,000, 8 > $140,000	5.3329	0.0281
Feeling Rushed (1 = every day, 6 = never)	2.1629	0.0174
Have Extra Time (1 = every day, 6 = never)	4.2035	0.0214
	**Weighted Proportion**	**S.E.**
Percent Female	0.5015	0.0065
Work >30 h/wk.	0.7249	0.0058
Children Present	0.4956	0.0065
Self-rated Mental Health (1 = E/VG/G)	0.9782	0.0022
Self-rated Health (1 = E/VG/G)	0.8596	0.0044
Self-rated Stress (1 = extremely/quite a bit)	0.1925	0.0052

Responses to questions on general perceptions of time use show respondents on average feel rushed a few times a week and feel themselves to have extra time about once a month. Approximately 98% of respondents report excellent, very good, or good mental health, 86% report excellent, very good, or good general health, and 19% report that most days are extremely or “quite a bit” stressful.

### 3.1 Regression analysis

Only models using the duration of meal preparation activities as the exposure variable are presented in this manuscript, with models using the count of meal preparation activities included in the Appendix. Generally, the direction and significance of the relationships between these primary independent variables and the response variables are consistent, but when differences occur, they are noted.

Before discussing the primary independent variables, it is worth noting that the directions of the control variables’ associations are generally as expected. Higher-income respondents report both feeling more rushed and also having more extra time, in addition to having better self-rated health and more self-rated stress. Female respondents report feeling more rushed and having less extra time than males, while also having higher levels of self-rated stress and better self-rated health. Older respondents report feeling rushed less often but also having less extra time. They also report having lower levels of self-rated health. When children are present, respondents report feeling more rushed and having less extra time, in addition to higher levels of self-rated mental health and stress. Finally, respondents who work 30 or more hours a week feel more rushed and have less extra time, in addition to having higher levels of self-rated mental health, health, and stress.

The results from the first models presented in Table 2 demonstrate the relationship between the duration of meal preparation activities to respondents' general perceptions of time. The category numbers represent the Likert scale responses of feeling rushed or having extra time, where 1 represents a respondent having the feeling every day and 6 represents never having the feeling. In order to make the odds ratios easier to interpret in these models, the duration in minutes has been divided by 10. Notably, feeling rushed is not significantly associated with the duration of meal preparation activities. However, both the unadjusted and adjusted models find a significant, positive association between respondents’ duration of meal preparation, and the feeling of having extra time. This shows that spending more time preparing meals is associated with a reduction in feelings of having extra time. Models run with the count of meal preparation activities (instead of duration) produce similar directions and magnitudes of odds ratios for three of the four models. However, the association between the count of meal preparation activities and feelings of having extra time was not significant in the relevant adjusted model (Table A.1).

**Table 2:** Results from proportional odds models exploring the association between general perceptions of time use and the duration of respondents’ meal preparation activities, in 10s of minutes. The outcome variables, feeling rushed and feelings of extra time, are represented by Likert scales, where 1 represents having the feeling frequently, and 6 represents never having the feeling.

	feeling rushed - unadjusted			feeling rushed - adjusted			extra time - unadjusted			extra time - adjusted		
	OR	2.50%	97.50%	OR	2.50%	97.50%	OR	2.50%	97.50%	OR	2.50%	97.50%
**Meal Prep Duration (in 10s of minutes)**	0.999	0.992	1.005	0.999	0.992	1.006	1.015**	1.009	1.022	1.009**	1.002	1.016
**HH Income Group**	–	–	–	0.977*	0.956	0.999	–	–	–	0.993^	0.972	1.015
**Female**	–	–	–	0.684**	0.621	0.754	–	–	–	1.462**	1.331	1.604
**Age Group**	–	–	–	1.212**	1.160	1.266	–	–	–	1.194**	1.145	1.245
**Children Present**	–	–	–	0.512**	0.463	0.566	–	–	–	1.404**	1.278	1.543
**Work >30 h/week**	–	–	–	0.421**	0.375	0.472	–	–	–	1.358**	1.207	1.528
**1| 2**	0.663**	0.623	0.704	0.284**	0.232	0.347	0.057**	0.051	0.065	0.140**	0.113	0.175
**2| 3**	2.639**	2.536	2.747	1.265*	1.214	1.318	0.265**	0.248	0.284	0.663**	0.620	0.709
**3| 4**	6.162**	5.781	6.568	3.164**	2.969	3.371	0.687**	0.649	0.727	1.756**	1.659	1.859
**4| 5**	10.184**	9.198	11.277	5.412**	4.890	5.988	1.229**	1.152	1.310	3.192**	2.991	3.406
**5| 6**	15.156**	13.051	17.599	8.216**	7.075	9.540	1.796**	1.654	1.950	4.717**	4.343	5.122

^ p < 0.1, * p < 0.05, ** p < 0.01.

The binary logistic regression models linking duration of meal preparation activities to self-rated health outcomes are presented in Table 3. As with the proportional odds models, meal preparation duration has been divided by 10 in order to make the odds ratios easier to interpret. For the unadjusted models, the duration of meal preparation activities is only significant when the dependent variable is d stress (recall 1 = extremely/quite a bit stressful), which shows a negative association. This relationship remains consistent and significant after adjusting for control variables and indicates that spending more time on meal preparation is associated with reporting lower levels of stress. The adjusted model focused on self-rated mental health shows a positive and significant (*p* < 0.1) relationship between duration of meal preparation activities, suggesting more time spent preparing meals is associated with improved levels of mental health. The adjusted self-rated health model shows no significant association with meal preparation duration.

**Table 3:** Results from logistic regression models exploring the link between self-rated health and well-being variables and the duration of respondents’ meal preparation activities, in 10s of minutes. The dependent variable self-rated stress is 1 with higher levels of stress and 0 for lower levels. The dependent variables self-rated mental health and self-rated health are 1 with better health and 0 with lower levels.

	Unadjusted Self-Rated Mental Health			Adjusted Self-Rated Mental Health			Unadjusted Self-Rated Health			Adjusted Self-Rated Health			Unadjusted Self-Rated Stress			Adjusted Self-Rated Stress		
	OR	2.50%	97.50%	OR	2.50%	97.50%	OR	2.50%	97.50%	OR	2.50%	97.50%	OR	2.50%	97.50%	OR	2.50%	97.50%
**Meal Prep Duration (in tens of minutes)**	1.031	0.990	1.074	1.035^	0.994	1.078	0.994	0.985	1.004	0.999	0.988	1.009	0.986*	0.976	0.997	0.985**	0.974	0.996
**HH Income Group**	–	–	–	0.985	0.891	1.088	–	–	–	1.123**	1.084	1.163	–	–	–	1.011	0.979	1.044
**Female**	–	–	–	1.205	0.747	1.943	–	–	–	1.175*	1.009	1.368	–	–	–	1.208**	1.050	1.391
**Age Group**	–	–	–	1.147	0.958	1.372	–	–	–	0.873**	0.813	0.936	–	–	–	0.962	0.903	1.026
**Children Present**	–	–	–	1.758*	1.112	2.779	–	–	–	1.075	0.913	1.265	–	–	–	1.260**	1.097	1.447
**Work >30 h/week**	–	–	–	3.903**	2.218	6.868	–	–	–	1.768**	1.490	2.098	–	–	–	1.527**	1.256	1.857
**Constant**	39.234**	29.200	52.716	9.243**	4.377	19.516	6.293***	5.755	6.881	2.925**	2.162	3.957	0.254**	0.234	0.276	0.155**	0.112	0.214
**Observations**	10,994			10,849			11,012			10,867			10,968			10,825		

^*p* < 0.1;*p < 0.05; **p < 0.01.

The direction and significance of associations in models using the count of meal preparation activities as the primary independent variable is consistent for the mental health and stress models (Table A.2). Interestingly, the relationship between self-rated health and the count of meal preparation episodes in the adjusted model shows a significant (*p* < 0.05) and positive relationship between the count of meal preparation activities and overall health, linking more meal preparation activities to better self-rated health.

### 3.2 Sequence analysis

As mentioned in the introduction, the duration of meal preparation activities affects and is affected by the type of activities that occur around meal preparation. In this subsection, the activities that surround evening (3 pm–9 pm) meal preparation activities are presented. Using all 7967 participants in the GSS Time Use Survey who reported preparing a meal during this window, a Sankey diagram illustrates the flow between the two preceding and two following activities, with meal preparation at the centre (Fig. 1). No weighting adjustment was used for this plot to allow for the direct flow between the five activities to be computed.

The figure shows some expected patterns. For example, approximately 50% of individuals engaged in an eating activity right after meal preparation, followed by participation in household chores, household maintenance or caring for another individual. However, breaking down the participation rates in the various categories by the response variables used in the previously reported regression models reveals interesting differences between those with different levels of self-rated health and perceptions of time.

In Table 4, Table 5, Table 6, the percentage of individuals, weighted with analytical weights, belonging to the nine super-activity categories in the two activities preceding and following meal preparation are reported. Percentages are stratified by binary classifications of the three outcome variables significantly associated with meal preparation time in the regression analyses. Table A.3 and Table A.4 display the results for the two outcome variables (feeling rushed and self-rated health) with no significant link to meal preparation time found in the presented regression models. A chi-square test is used to assess whether the distributions across activity categories are the same at each preceding and following activity.

**Table 4:** Distribution of activities by Canadian adults who prepared a meal in the evening participated in preceding and following meal preparation, stratified by replies to whether they feel as though they have extra time (more = everyday, a few times a week, and once a week; less = about once a month, less than once a month, and never).

Activity Category	Extra Time									
	2 Act. Before**		1 Act. Before**		Meal Prep		1 Act. After**		2 Act. After**	
	More	Less	More	Less	More	Less	More	Less	More	Less
Meal preparation	3.00%	2.95%	0.32%	0.29%	100.00%	100.00%	3.61%	3.65%	3.00%	3.01%
Sleeping, napping, resting, own personal care	7.84%	7.52%	8.60%	8.99%	0.00%	0.00%	4.44%	4.49%	16.24%	14.60%
Eating, drinking	5.93%	6.82%	1.58%	1.87%	0.00%	0.00%	48.40%	51.12%	6.92%	8.44%
Travel, going from place to place	22.11%	21.50%	32.98%	33.97%	0.00%	0.00%	5.75%	4.59%	5.63%	5.16%
Paid work activities, study	14.93%	17.35%	4.88%	4.33%	0.00%	0.00%	1.64%	1.71%	3.06%	2.17%
HH Chores, or maintenance, caring for others	15.42%	17.56%	16.10%	21.20%	0.00%	0.00%	8.04%	13.52%	26.23%	30.86%
Shopping for goods or services	12.20%	11.16%	5.41%	5.02%	0.00%	0.00%	2.95%	2.32%	4.09%	3.43%
Socializing or communicating; civic, religious, organized activities; sports, exercise, leisure	18.27%	14.92%	29.74%	23.98%	0.00%	0.00%	25.11%	18.33%	34.06%	31.78%
Uncodeable, other	0.29%	0.22%	0.39%	0.35%	0.00%	0.00%	0.05%	0.26%	0.77%	0.55%

Significance reported for chi-square tests comparing the distribution between the two categories (^*p* < 0.10, *p < 0.05, **p < 0.01).

**Table 5:** Distribution of activities by Canadians adults who prepared a meal in the evening participated in preceding and following meal preparation, stratified by self-rated mental health (excellent, very good, or good vs. fair or poor).

Activity Category	Self-Rated Mental Health									
	2 Act. Before		1 Act. Before		Meal Prep		1 Act. After		2 Act. After**	
	E/VG/G	F/P	E/VG/G	F/P	E/VG/G	F/P	E/VG/G	F/P	E/VG/G	F/P
Meal preparation	2.92%	2.80%	0.34%	0.00%	100.00%	100.00%	3.75%	2.59%	2.66%	5.57%
Sleeping, napping, resting, own personal care	7.41%	8.76%	8.60%	8.93%	0.00%	0.00%	4.20%	6.49%	15.22%	15.89%
Eating, drinking	6.32%	7.14%	1.80%	1.74%	0.00%	0.00%	50.08%	50.04%	8.11%	6.15%
Travel, going from place to place	22.12%	18.37%	33.52%	35.29%	0.00%	0.00%	4.97%	5.62%	5.47%	4.47%
Paid work activities, study	16.17%	18.93%	4.51%	3.55%	0.00%	0.00%	1.62%	1.82%	2.33%	4.45%
HH Chores, or maintenance, caring for others	16.99%	15.95%	19.48%	18.18%	0.00%	0.00%	11.25%	11.67%	29.03%	28.25%
Shopping for goods or services	11.69%	11.03%	5.24%	5.55%	0.00%	0.00%	2.65%	2.01%	3.82%	2.80%
Socializing or communicating; civic, religious, organized activities; sports, exercise, leisure	16.11%	16.93%	26.16%	26.47%	0.00%	0.00%	21.29%	19.75%	32.76%	31.67%
Uncodeable, other	0.27%	0.08%	0.35%	0.28%	0.00%	0.00%	0.18%	0.02%	0.60%	0.74%

Significance reported for chi-square tests comparing the distribution between the two categories (^*p* < 0.10, *p < 0.05, **p < 0.01).

**Table 6:** Distribution of activities by Canadian adults who prepared a meal in the evening participated in preceding and following meal preparation, stratified by self-rated stress (not at all, not very, a bit vs. quite a bit or extremely).

Activity Category	Self-Rated Stress									
	2 Act. Before**		1 Act. Before**		Meal Prep		1 Act. After**		2 Act. After**	
	NAA/NV/AB	QAB/E	NAA/NV/AB	QAB/E	NAA/NV/AB	QAB/E	NAA/NV/AB	QAB/E	NAA/NV/AB	QAB/E
Meal preparation	3.05%	2.01%	0.36%	0.04%	100.00%	100.00%	3.85%	2.43%	2.75%	4.03%
Sleeping, napping, resting, own personal care	7.68%	6.56%	8.79%	7.44%	0.00%	0.00%	4.65%	3.32%	15.74%	12.87%
Eating, drinking	6.64%	4.76%	1.87%	1.37%	0.00%	0.00%	49.66%	52.48%	8.03%	7.33%
Travel, going from place to place	21.69%	22.30%	32.17%	42.72%	0.00%	0.00%	4.93%	5.80%	5.13%	6.86%
Paid work activities, study	14.83%	25.92%	4.21%	5.80%	0.00%	0.00%	1.51%	2.52%	2.20%	4.52%
HH Chores, or maintenance, caring for others	16.91%	17.17%	19.44%	18.85%	0.00%	0.00%	10.77%	13.93%	28.46%	32.04%
Shopping for goods or services	11.95%	9.52%	5.27%	5.07%	0.00%	0.00%	2.65%	2.30%	3.88%	2.78%
Socializing or communicating; civic, religious, organized activities; sports, exercise, leisure	17.00%	11.55%	27.56%	18.33%	0.00%	0.00%	21.89%	17.07%	33.13%	29.23%
Uncodeable, other	0.26%	0.22%	0.34%	0.39%	0.00%	0.00%	0.10%	0.15%	0.67%	0.33%

Significance reported for chi-square tests comparing the distribution between the two categories (^*p* < 0.10, *p < 0.05, **p < 0.01).

The null hypothesis (that the distributions are equal) is not rejected for activities preceding and the activity directly following meal preparation when stratifying by self-rated mental health status (Table 5). However, the second activity after meal preparation is significant at the *p* < 0.01 level. For both self-rated stress and reporting feelings of having more extra time (Table 4, Table 6), the null hypothesis is rejected across all preceding and following activities at the *p* < 0.01 level.

Prior to the evening meal preparation activity, travel, work and study, chores, and socializing and leisure are common (Fig. 1). A larger proportion of individuals with higher levels of stress traveled immediately prior to meal preparation, compared to their counterparts with lower stress levels (42.7% vs 32.3%, respectively) (Table 6). Meanwhile, a greater number of participants with lower stress levels engaged in socializing and leisure two activities prior to meal preparation (17.0%), compared with participants with higher stress levels (11.6%) (Table 6). Similarly, a greater percentage (ranging from 2.3% to 5.8%) of those reporting more extra time participated in socializing and leisure before and after meal preparation (Table 4).

Directly following meal preparation, eating is clearly identified as the most common activity. Chores and care activities, socializing and leisure, and sleeping or personal care activities most commonly account for the second activity following meal preparation (Fig. 1). A greater number of participants with higher self-rated levels of stress did chores and care activities following meal preparation (3.2–3.5% more than those with lower levels of stress in the two activities that follow) (Table 6). Likewise, a lower percentage of those reporting more extra time (2.1%) participated in chores and care activities both before and after meal preparation compared to those reporting less extra time (5.5%) (Table 4).

Given the results of the chi-square test, the differences in activity participation between those with higher and lower self-rated mental health are small and mostly insignificant.

## 4 Discussion

Using two methodological approaches on a representative sample of Canadian adults, the analyses presented in this paper demonstrate that further exploration into the role meal preparation activities play in health and well-being is warranted. The regression analyses show there are significant associations between the amount of time spent on meal preparation in a day and higher self-rated mental health, lower self-rated stress, and reduced feelings of having extra time. The analysis of the sequences of activities surrounding evening meal preparation, stratified by these outcome variables, helps connect measures of health and well-being with the context in which participants engage in meal preparation. For example, those reporting higher levels of stress or feeling like they have little extra time are more likely to participate in chores and care activities before and after evening meal preparation.

Taken together, these results are a step forward in establishing that time spent on preparing meals is a significant factor in a person's health and well-being, and that there are complex reasons that people spend more or less time on meal preparation activities. They also inspire a number of new questions related to limitations of the current study's dataset and analytical approach.

A first question that arises is: to what extent is there a causal relationship between time spent on meal preparation and health outcomes, and if there is a causal link, what is its direction? For example, those with more self-rated stress also report spending less time on meal preparation (Table 3). Does increased stress lead to reductions in time spent preparing meals, or does not having time for preparing meals result in high stress levels? The results from the sequence analysis that show people with higher levels of stress are more likely to participate in unpaid labor activities like childcare and household chores potentially offer some insights into this question. Higher percentages of those with more stress participated in household labor surrounding evening meal preparation. This may point towards those with higher stress having less time available for meal preparation.

However, this link is complicated by the findings related to those who reported having more extra time. Those with less extra time spent more time on meal preparation but, similar to those with more stress, were more likely to participate in household labour activities before and after an evening meal preparation. The reason for this could be related to the associations and interactions between self-rated stress and perceptions of time use. Future work is needed to systematically examine these more complex associations, as well as the types, durations, and sequences of activities that occur around meal preparation to firmly establish the potential causal pathways described here.

A second question in need of exploration is: what role do social and spatial contexts play in the time spent on meal preparation and other food-related activities? For many, meal preparation is taken on by one individual, and the results shared with all household members. In a partnered household, the division of labour could be such that one individual could spend no time on meal preparation while the other spends significant time on this activity. Unfortunately, the dataset used in this study was not designed to concurrently examine time use for partnered individuals, but such an analysis would prove useful in unpacking the associations presented in this paper. Like social context, the geographic context of an individual will influence their time use. For example, those with a car may spend less time in transit between activities, thus allowing more time to spend on both discretionary and non-discretionary activities than a person who relies on public transportation. Again, data on individuals’ geographic patterns were not available in the dataset used here, but past work has demonstrated the utility of taking such an approach (Kwan, 1999).

Finally, this study's reliance on a one-day, country-level time use survey should be noted. Variations at regional scales (e.g., province to province, or suburban areas to urban ones) are not examined, and as with any large survey, sampling errors are unavoidable. That said, the large sample size and use of weights in the analyses presented in this paper provide for results which are robust.

### 4.1 Conclusion

This paper has established that both time spent on meal preparation and the sequences of activities that surround evening meal preparation are significantly linked to numerous health-related variables. Further work in this area will serve to add to the evidence base used by public health researchers and policy makers to develop interventions that aim to improve diet, nutrition, and, ultimately, overall health and well-being.

## Ethical statement

Per guidelines from the University of Toronto Research Ethics Board, no ethics review for this analysis was required, as we used data that is “publicly available through a mechanism set out by legislation or regulation and that is protected by law.”

https://research.utoronto.ca/ethics-human-research/activities-exempt-human-ethics-review.

## Author contributions

*Michael J. Widener* – Conceptualization; Data curation; Formal analysis; Funding acquisition; Investigation; Methodology; Project administration; Resources; Software; Supervision; Validation; Visualization; Writing - original draft; Writing - review & editing.

*Linda Ren* - Conceptualization; Data curation; Formal analysis; Visualization; Writing - review & editing, *Chloe C. Astbury* - Conceptualization; Methodology; Writing - review & editing.

*Lindsey G. Smith* - Conceptualization; Methodology; Writing - review & editing.

*Tarra Penney* - Conceptualization; Methodology; Writing - review & editing.

## Funding

Funding: This research was undertaken, in part, thanks to funding from the Canada Research Chairs Program (Widener).

## Declaration of competing interest

None.

## Figures and Tables

**Table A.1:** Results from proportional odds models exploring the link between general perceptions of time use and the count of respondents' meal preparation activities.

	feeling rushed - unadjusted			feeling rushed - adjusted			extra time - unadjusted			extra time - adjusted		
	OR	2.50%	97.50%	OR	2.50%	97.50%	OR	2.50%	97.50%	OR	2.50%	97.50%
**# Meal Prep Act.**	1.010	0.967	1.056	1.022	0.975	1.071	1.060*	1.012	1.111	1.008	0.961	1.057
**HH Income Group**				0.977*	0.956	0.999	–	–	–	0.993	0.972	1.015
**Female**				0.678**	0.615	0.748	–	–	–	1.480**	1.347	1.626
**Age Group**				1.211**	1.159	1.265	–	–	–	1.196**	1.148	1.247
**Children Present**				0.508**	0.459	0.562	–	–	–	1.419**	1.291	1.558
**Work >30 h/week**				0.423**	0.378	0.474	–	–	–	1.342**	1.193	1.510
**1| 2**	0.674**	0.628	0.723	0.290**	0.237	0.355	0.057**	0.050	0.065	0.137**	0.109	0.171
**2| 3**	2.685**	2.580	2.794	1.296*	1.244	1.350	0.262**	0.245	0.281	0.645**	0.603	0.690
**3| 4**	6.268**	5.881	6.681	3.240**	3.041	3.453	0.679**	0.642	0.718	1.708**	1.613	1.807
**4| 5**	10.359**	9.356	11.470	5.542**	5.008	6.133	1.213**	1.137	1.294	3.102**	2.907	3.310
**5| 6**	15.416**	13.275	17.901	8.413**	7.245	9.770	1.772**	1.632	1.924	4.583**	4.220	4.977

^*p* < 0.1, *p < 0.05, **p < 0.01.

**Table A.2:** Results from logistic regression models exploring the link between self-rated health and well-being variables and the count of respondents' meal preparation activities.

	Unadjusted Self-Rated Mental Health			Adjusted Self-Rated Mental Health			Unadjusted Self-Rated Health			Adjusted Self-Rated Health			Unadjusted Self-Rated Stress			Adjusted Self-Rated Stress		
	OR	2.50%	97.50%	OR	2.50%	97.50%	OR	2.50%	97.50%	OR	2.50%	97.50%	OR	2.50%	97.50%	OR	2.50%	97.50%
**# Meal Prep Activities**	1.211	0.949	1.546	1.234^	0.976	1.561	1.048	0.976	1.125	1.091*	1.011	1.177	0.920*	0.861	0.982	0.908**	0.847	0.975
**HH Income Group**	–	–	–	0.985	0.893	1.087	–	–	–	1.123**	1.084	1.163	–	–	–	1.011	0.979	1.044
**Female**	–	–	–	1.184	0.732	1.916	–	–	–	1.140^	0.977	1.330	–	–	–	1.216**	1.056	1.401
**Age Group**	–	–	–	1.146	0.959	1.37	–	–	–	0.87**	0.811	0.933	–	–	–	0.962	0.902	1.025
**Children Present**	–	–	–	1.743*	1.111	2.734	–	–	–	1.050	0.892	1.235	–	–	–	1.263**	1.100	1.450
**Work >30 h/week**	–	–	–	3.902**	2.227	6.835	–	–	–	1.813**	1.528	2.152	–	–	–	1.523**	1.253	1.852
**Constant**	37.257**	26.559	52.266	8.837**	4.182	18.675	5.833**	5.257	6.473	2.697**	1.995	3.648	0.260**	0.236	0.286	0.159**	0.115	0.221
**Observations**	10,994			10,849			11,012			10,867			10,968			10,825		

^*p* < 0.1;*p < 0.05; **p < 0.01.

**Table A.3:** Distribution of activities Canadians who prepared a meal in the evening participated in preceding and following meal preparation, stratified by replies to how often they feel rushed (more = everyday, a few times a week, and once a week; less = about once a month, less than once a month, and never).

Activity Category	Feeling Rushed									
	2 Act. Before**">**		1 Act. Before**">**		Meal Prep		1 Act. After**">**		2 Act. After**">**	
	More	Less	More	Less	More	Less	More	Less	More	Less
Meal preparation	2.96%	3.02%	0.24%	0.54%	100.00%	100.00%	3.54%	4.02%	3.06%	2.75%
Sleeping, napping, resting, own personal care	7.34%	8.65%	8.41%	10.35%	0.00%	0.00%	4.78%	3.28%	15.02%	16.17%
Eating, drinking	5.81%	8.95%	1.62%	2.28%	0.00%	0.00%	49.55%	51.93%	7.59%	8.76%
Travel, going from place to place	22.16%	20.18%	37.33%	19.68%	0.00%	0.00%	5.56%	3.14%	5.75%	3.86%
Paid work activities, study	19.41%	5.32%	5.26%	1.91%	0.00%	0.00%	1.96%	0.64%	2.99%	0.84%
HH Chores, or maintenance, caring for others	16.20%	18.59%	20.09%	16.13%	0.00%	0.00%	12.35%	7.73%	30.40%	24.25%
Shopping for goods or services	11.53%	11.71%	5.08%	5.47%	0.00%	0.00%	2.65%	2.29%	3.82%	3.21%
Socializing or communicating; civic, religious, organized activities; sports, exercise, leisure	14.31%	23.50%	21.63%	43.24%	0.00%	0.00%	19.44%	26.79%	30.76%	39.45%
Uncodeable, other	0.29%	0.08%	0.36%	0.40%	0.00%	0.00%	0.18%	0.18%	0.61%	0.72%

Significance reported for chi-square tests comparing the distribution between the two categories (^*p* < 0.10, *p < 0.05, **p < 0.01).

**Table A.4:** Distribution of activities Canadians who prepared a meal in the evening participated in preceding and following meal preparation, stratified by self-rated health (excellent, very good, or good vs. fair or poor)

Activity Category	Self-Rated Health									
	2 Act. Before*">*		1 Act. Before**">**		Meal Prep		1 Act. After*">*		2 Act. After^">^	
	E/VG/G	F/P	E/VG/G	F/P	E/VG/G	F/P	E/VG/G	F/P	E/VG/G	F/P
Meal preparation	2.88%	3.05%	0.34%	0.09%	100.00%	100.00%	3.72%	3.18%	3.04%	2.01%
Sleeping, napping, resting, own personal care	7.26%	9.30%	8.56%	9.09%	0.00%	0.00%	4.42%	4.53%	15.10%	16.82%
Eating, drinking	6.27%	7.12%	1.87%	1.31%	0.00%	0.00%	49.98%	50.59%	8.00%	7.37%
Travel, going from place to place	22.19%	18.97%	33.56%	34.52%	0.00%	0.00%	5.20%	3.92%	5.45%	4.87%
Paid work activities, study	16.79%	14.11%	4.82%	1.78%	0.00%	0.00%	1.80%	0.63%	2.69%	1.39%
HH Chores, or maintenance, caring for others	16.91%	16.94%	19.50%	18.28%	0.00%	0.00%	11.40%	10.49%	28.72%	30.54%
Shopping for goods or services	11.41%	13.06%	5.37%	4.53%	0.00%	0.00%	2.66%	2.11%	3.82%	3.05%
Socializing or communicating; civic, religious, organized activities; sports, exercise, leisure	16.00%	17.41%	25.64%	30.05%	0.00%	0.00%	20.64%	24.54%	32.55%	33.28%
Uncodeable, other	0.28%	0.04%	0.34%	0.35%	0.00%	0.00%	0.20%	0.00%	0.61%	0.68%

Significance reported for chi-square tests comparing the distribution between the two categories (^*p* < 0.10, *p < 0.05, **p < 0.01).
